# Study of Brain Cells in Neurodegenerative Diseases: Raman Microspectroscopy and Scanning Ion-Conductance Microscopy

**DOI:** 10.17691/stm2025.17.1.03

**Published:** 2025-02-28

**Authors:** K.I. Morozova, E.Yu. Parshina, T.A. Kazakova, A.I. Yusipovich, O.V. Slatinskaya, A.R. Brazhe, I.A. Grivennikov, N.A. Brazhe, G.V. Maksimov

**Affiliations:** Junior Researcher, Department of Biophysics, Faculty of Biology; Lomonosov Moscow State University, 1 Leninskiye Gory, Moscow, 119991, Russia; PhD, Senior Researcher, Department of Biophysics, Faculty of Biology; Lomonosov Moscow State University, 1 Leninskiye Gory, Moscow, 119991, Russia; Engineer, Department of Biophysics, Faculty of Biology; Lomonosov Moscow State University, 1 Leninskiye Gory, Moscow, 119991, Russia; PhD, Senior Researcher, Department of Biophysics, Faculty of Biology; Lomonosov Moscow State University, 1 Leninskiye Gory, Moscow, 119991, Russia; PhD, Junior Researcher, Department of Biophysics, Faculty of Biology; Lomonosov Moscow State University, 1 Leninskiye Gory, Moscow, 119991, Russia; PhD, Senior Researcher, Department of Biophysics, Faculty of Biology; Lomonosov Moscow State University, 1 Leninskiye Gory, Moscow, 119991, Russia; Senior Researcher, Neuron-Glial Dynamics Group; Shemyakin and Ovchinnikov Institute of Bioorganic Chemistry of the Russian Academy of Sciences, 16/10 Miklukho-Maklaya St., Moscow, 117997, Russia; DSc, Professor, Chief Researcher, Laboratory of Molecular Neurogenetics and Innate Immunity; National Research Center “Kurchatov Institute”, 1 Akademika Kurchatova Square, Moscow, 123182, Russia; PhD, Leading Researcher, Department of Biophysics, Faculty of Biology; Lomonosov Moscow State University, 1 Leninskiye Gory, Moscow, 119991, Russia; Senior Researcher, Redox Neurobiology Group; Shemyakin and Ovchinnikov Institute of Bioorganic Chemistry of the Russian Academy of Sciences, 16/10 Miklukho-Maklaya St., Moscow, 117997, Russia; DSc, Professor, Department of Biophysics, Faculty of Biology; Lomonosov Moscow State University, 1 Leninskiye Gory, Moscow, 119991, Russia; Professor, Department of Physical Materials; National University of Science and Technology “MISIS”, 4 Leninsky Prospect, Moscow, 119049, Russia

**Keywords:** Raman spectroscopy, surface-enhanced Raman spectroscopy, plasmonic silver nanostructures, scanning ionconductance microscopy, neuron, astrocyte, pluripotent stem cells

## Abstract

**The aim of the study** was to identify differences in the structure of the neuronal process network as well as the composition and functional state of cells by studying the bodies and processes of rat brain neurons and astrocytes obtained from pluripotent stem cells of healthy donors and patients with hereditary Parkinson’s disease by using a complex of modern high-precision methods such as Raman microspectroscopy, surface-enhanced Raman microspectroscopy, and scanning ion-conductance microscopy.

**Materials and Methods:**

By using Raman spectroscopy and scanning ion-conductance microscopy, the researchers studied the morphology and state of molecules in rat brain neurons and astrocytes induced from pluripotent stem cells of healthy donors and patients with hereditary Parkinson’s disease.

**Results:**

The researchers established that typical bands of Raman and surface-enhanced Raman spectra of neurons and astrocytes allowed studying the distribution and conformation of a series of biological molecules (proteins, lipids, cytochromes) in healthy and unhealthy states. It was shown that in Parkinson’s disease, there was a decrease in the protein content and an increase in the proportion of reduced cytochromes in the respiratory chain of astrocyte mitochondria. When comparing the morphology of astrocyte bodies and processes, it was established that the height and cross-sectional area of astrocyte processes obtained from cells of patients with hereditary Parkinson’s disease were significantly greater than in healthy patients.

**Conclusion:**

The developed approach to recording the distribution and conformation of molecules in neurons and astrocytes, as well as to studying the morphology of astrocyte processes allows diagnosing the functional state of cells and investigating the mechanism of the Parkinson’s disease pathogenesis.

## Introduction

Raman spectroscopy (RS), or combination spectroscopy, is a sensitive and highly selective technique for studying conformational changes and the redox state of molecules *in vitro* and *in vivo*. RS allows one studying living cells in native conditions without any damage and preliminary preparation as well as obtaining unique information not only about the molecular composition, but also about the functional state of cells, based on data about the vibrational structure of biological molecules [1–3]. One of the RS varieties, surface-enhanced Raman spectroscopy (SERS), is a sensitive and universal tool for chemical and biochemical diagnostics [4–8]. This technique applies the effect of the surface plasmonic resonance and unique modes of molecular vibrations to enhance the signal and allows identifying the structure to the level of single molecules. In the last decade, it has been demonstrated that SERS can also be used to study individual cells, bacteria, and viruses in their natural environment [9–11]. High sensitivity of this technique allows studying the slightest changes in the biochemical composition of cells and provides an unprecedented chance to monitor the dynamics of individual cells functional state [12–14]. However, when using SERS, researchers note significant difficulties, in particular those related to stability and reproducibility of nanostructured substrates and the recorded SERS signals, with the interaction of the substrate surface with the membrane of living cells, with the lateral mobility of cells on the substrate and the cell contact with plasmonic nanostructures [4, 5, 15]. Thus, studies in which RS and SERS are used to study native animal and human cells in their normal state and in various pathologies are of major importance.

A serious neurodegenerative pathology accompanied by the brain cells damage, in particular changes in the morphology and functioning of neurons and astrocytes, is Parkinson’s disease (PD). The main link in the molecular pathogenesis of PD is the synthesis and formation of neurotoxic aggregates of the α-synuclein protein, the aggregation of which is stimulated not only by point mutations and multiplication of the gene encoding α-synuclein, *SNCA*, but also by increasing oxidative stress and mitochondrial dysfunction [16]. This results in accumulation of modified “inappropriate” proteins, which impact the morphology of synapses, neuronal processes, and astrocytes.

It is known that astrocytes are important for the formation of synapses, maintaining the balance of neurotransmitters, potassium, and pH, as well as for developing contacts with brain capillaries [17]. Astrocytes regulate the O_2_ flow from blood vessels into brain tissue [18] and provide metabolic support to neurons by supplying them with lactate and substrates for the synthesis of neurotransmitters [17]. They are also involved in the pathogenesis of various neurodegenerative diseases, including PD [19], and here changes in their morphology and functional activity are seen [19]. This process is called reactive astrogliosis and is characterized by an increase in the production of glial fibrillary acidic protein (GFAP) [20]. Such changes are aimed at the CNS damage reduction, but in case of chronic reactive astrogliosis they result in an increase in the production of reactive oxygen species (ROS) as well as in the release of proinflammatory mediators, which causes neuronal damage [20]. An increase in ROS production by astrocytes and oxidative stress in PD is associated with both impaired mitochondrial functioning and impaired oxidative stress protective systems [21]. Moreover, astrocytes are important for utilization of α-synuclein, which arrives from neurons [19, 20]. In the brain, neuron disorder is initiated by changes in the astrocytes functioning, which provide neuronal metabolism.

To study the cellular mechanisms of development of neurodegenerative brain diseases associated with PD, one requires development and implementation of new effective techniques for studying both the morphology of neurons and astrocytes and the redox state of the components of the respiratory electron transport chain (ETC) of mitochondria, which determines the synthesis of adenosine triphosphate (ATP). As mentioned above, PD is accompanied by changes in the morphology of nerve cell processes and the synapse area. A modern technique for the maximum non-invasive study of the morphology of living cells in culture is scanning ionconductance microscopy (SICM) [22–25]. It is one of the probe microscopy techniques, where a micropipette is used as a probe, and resistance serves as a feedback signal. Approaching the micropipette to the cell surface causes an increase in resistance (a decrease in conductivity), while, unlike such a wide-spread technique as atomic force microscopy, the probe does not directly touch the cell surface, thus avoiding damage to its surface. This technique allows to accurately and non-invasively assess cell geometry, including its fine processes, which is important for studying the structure of neurons and astrocytes in PD.

Currently, studies using induced pluripotent stem cells (iPSCs) gain major interest. iPSCs are stem cells that were obtained from somatic differentiated cells (for instance, fibroblasts) using various molecular manipulations. Such iPSCs can be directed along another developmental pathway, to get, for example, neurons, astrocytes, etc. In neurobiology, iPSCs are actively used for differentiation into a specific type of nerve cells as well as for studying the development of neuropathologies at the cellular level.

There are several ways to transform somatic cells into iPSCs: for example, using skin fibroblasts followed by their conversion into iPSCs using a standard protocol — CytoTune-iPS 2.0 Sendai Reprogramming Kit (Thermo Fisher Scientific, USA) [26]. In the laboratory, the obtained iPSCs are differentiated into neurons or astrocytes by using a combination of various growth factors and metabolites [26]. Using iPSCs induced from somatic cells of the diseased being carriers of a hereditary disease gene, one can study the development of such a disease at the cellular level.

**The aim of the study** was to identify differences in the structure of the neuronal process network, the composition and functional state of cells by studying the bodies and processes of rat brain neurons and astrocytes obtained from pluripotent stem cells of healthy donors and patients with hereditary Parkinson’s disease by using a complex of modern high-precision techniques — Raman microspectroscopy, surfaceenhanced Raman microspectroscopy, and scanning ionconductance microscopy.

## Materials and Methods

### Rat brain neuron culture

Primary culture of cerebellar granule cells was prepared using 2–4-dayold Wistar–Kyoto rats. Decapitation was conducted in accordance with ethical requirements for working with animals. All manipulations with animals were carried out in accordance with the standards specified in the Guide for the Care and Use of Laboratory Animals (National Research Council, 2011); with Principles of Good Laboratory Practice — the national standard of the Russian Federation GOST 33044–2014; with the ethical principles of the European Convention for the Protection of Vertebrate Animals used for Experimental and Other Scientific Purposes (Strasbourg, 2006). The study protocol was approved by the Ethics Committee of the Biological Faculty of the Lomonosov Moscow State University (protocol No.82-O dated June 08, 2017).

All procedures with cells were conducted in sterile conditions. The cerebellar tissue was washed in cold calcium- and magnesium-free Hanks’ solution (Gibco, USA) with 0.04% NaHCO_3_, cleared of vessels, films, brainstem tissue, and other components, grounded, after which the cell suspension was placed for 15 min in 0.05% trypsin with 0.02% EDTA (Gibco, USA) at the temperature of 37°C. After incubation, the cells were placed for 2–3 min in 2–3 ml of bovine serum to stop the reaction, and then trypsin was washed twice with standard Hanks’ solution with phenol red and MEM (minimal essential medium) culture medium, both produced by Gibco (USA). The cells were then dispersed in fresh MEM until a homogeneous suspension was obtained, which was centrifuged for 2 min at 3000 rpm. The precipitated cells were resuspended in NBM (neurobasal medium) (Gibco, USA) containing 2% Supplement B–27; 0.5 mM GlutaMax; 100 U/ml penicillin/streptomycin (all produced by Gibco, USA), and 20 mM KCl (Sigma, USA). Neurons were counted in a Goryaev chamber; their usually amounted to 1.0– 3.5×10^6^ cells/ml. 100–150 μl of the cell suspension were transferred to Petri dishes preincubated with polyornithine (Sigma, USA) to improve cell adhesion to their surface. Cultivation was conducted in NBM for 5–7 days in a CO_2_ incubator at 37°C, 5% CO_2_ and a relative humidity of 98%.

### Differentiated astrocyte cultures

Differentiated astrocytes were received from iPSCs obtained from healthy donors and patients with hereditary PD (G2019S mutation in the *LRRK2* gene). Further differentiation of iPSCs to neuronal precursors was carried out using the earlier described technique [27]. Glial cells were from neuronal precursors, the protocol described in detail in [28] was used. The resulting glial cell cultures were characterized by the expression levels of a number of neuroglial differentiation genes [28].

### Raman microspectroscopy

The distribution and conformation of proteins and lipids in the cytoplasm and the relative content of reduced cytochromes in the mitochondria ETC were studied using a confocal Raman spectrometer NTEGRA Spectra (NT-MDT, Russia) combined with an inverted Olympus microscope (Olympus, Japan) with laser excitation and a wavelength of 532 nm, a laser power at the output of the objective of 1 mW and a 20× NA0.40 objective. One spectrum was recorded for 60 s. The conditions for recording the Raman spectra were selected so that the illumination did not impact the intensity of the Raman signal from the sample for at least 6–8 repeated laser illuminations of astrocytes during 60 s.

### Surface-enhanced Raman spectroscopy

The study of lipids and proteins of the plasma membrane of thin processes was conducted by means of SERS using silver plasmonic nanostructured surfaces (SERS nanostructures). The synthesis of SERS nanostructures was performed as described in [4, 5, 15]. Nanostructured surfaces were obtained using ultrasonic spraying of an ammonia solution of silver oxide (for 60 min) on pre-cleaned cover glasses heated to 270°C. After preparation, the nanostructured surfaces were kept in a dark place at room temperature for 7 days. To get an enhanced Raman signal from the plasma membrane components of thin neuronal processes, the selected neuronal processes were covered with a small 3×3 mm piece of glass with SERS nanostructures; here, the nanostructured surface was oriented relative to the 20× NA0.40 objective of the NTEGRA Spectra confocal Raman spectrometer (NT-MDT, Russia). The experiments were conducted using laser excitation with a wavelength of 532 nm and a power of up to 1 mW per recording area. The SERS spectra accumulation time was 60 s.

The baseline correction in the RS and SERS spectra was performed using the open source Pyraman software (https://github.com/abrazhe/pyraman). Graphs were prepared in OriginPro 2022. Signal processing included baseline subtraction and signal smoothing for 10 points.

### Scanning ion-conductance microscopy

To study the morphology of the processes and the distribution of cytoplasm in the cell, the researchers used a scanning ion-conductance microscope created based on an inverted optical microscope Eclipse Ti-2 (Nikon, Japan), which was placed on an STable vibration-isolating table (Supertech Instruments, Hungary) and consisted of the following functional systems: a Mechanical stand scanning platform, positioning and piezo control systems, a Universal Controller feedback control system (all systems are manufactured by ICAPPIC Limited, UK), as well as MultiClamp 700B and Digidata 1550B input-output interfaces (Axon Instruments, USA). The authors used this scanning ion-conducting microscope in the hopping mode, the scanning field sized 40×40 μm, the diameter of the inner hole of the pipette was 50 nm, the setpoint (the value of the current drop during topography scanning) was 0.5%, an image scanning duration was 1.5–2.0 min. For image taking, neurons were placed in Hanks’ solution, prescan was performed during scanning; while scanning, each square was first assessed for the height of 4 corners of the square, and then the inside of the selected area was scanned. When scanning astrocyte bodies, this value was about 6000 nm, when scanning processes — 2000 to 4000 nm.

### Statistical processing of results

The results were processed in the demo version of the Prism 10 software (GraphPad, USA). In the figures, the data are shown as a median with an interquartile range. The statistical significance of differences was assessed using the Mann–Whitney test, p<0.05.

## Results and Discussion

### Study of the redox state of mitochondrial cytochromes, protein-lipid composition of the cytoplasm and plasma membrane of cerebellar neurons

**Figure 1.**
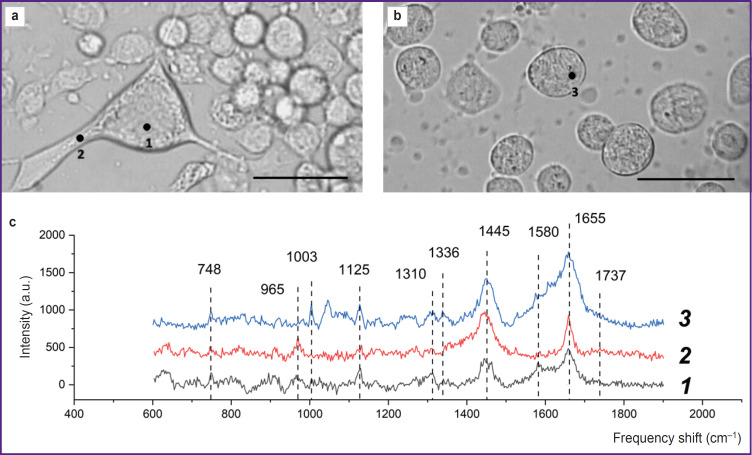
Typical Raman spectra from neurons of the rat cerebellum: (a) neuron with processes; (b) spherical neurons; bar — 10 μm; (c) Raman spectra taken from the areas of the neuron body (*1*), neuron process (*2*), and spherical neuron body (*3*)

RS was used to study the distribution and conformation of proteins and lipids in the bodies, and processes of neurons in a rat cerebellar neuron culture were studied. During the study, Raman spectra were obtained from 9 neurons: without processes and with 1 to 3 clearly distinguishable processes (Figure 1).

A neuron cell with processes (Figure 1 (a)) and spectra (Figure 1 (c)) recorded in the cell body (*1*) and on the process (*2*) are shown. The Raman spectrum contains bands typical of protein bond vibrations: 1655 cm^–1^ (Amide I), which corresponds to vibrations of the peptide bond in protein molecules with alpha structures; 1445 cm^–1^ — vibrations of methylene groups in lipid and protein molecules (with a significant contribution from lipids); 1003 cm^–1^ — vibrations of the aromatic ring in phenylalanine residues of proteins. The Raman spectrum bands with maximum values of 748 and 1125 cm^–1^ correspond to vibrations of bonds in the heme of reduced cytochromes *c* and *b*, with the peak at 748 cm^–1^ most likely being determined by vibrations of the heme of reduced cytochromes *c* and *c_1_*, and the band at 1125 cm^–1^ — by vibrations of the bonds of the hemes of reduced cytochromes *b_high_* and *b_low_* of complex III of the ETC. The Raman spectrum band with the maximum peak position of 1336 cm^–1^ is the result of hemes vibrations of only reduced cytochromes *b* of complex III and, thus, is a qualitative indicator of the number of reduced cytochromes *b_high_* and *b_low_* of complex III of the ETC. The Raman spectrum band with a peak at 1310 cm^–1^ is the result of summing up two peaks with maximum positions at 1300 and 1315 cm^–1^, which are characteristic of reduced cytochromes *b* and *c*, respectively. Moreover, a characteristic Raman spectrum band of cytochromes (“cytochrome peak”) is the maximum at 1580 cm^–1^, which shows itself as a shoulder at the peak with a maximum position at 1655 cm^–1^.

It should be noted that sufficient intensity and good resolution of the Raman signal can be obtained only from large neuron processes. The Raman spectra from different areas of the neuron provides that the parameters of the Raman spectrum in the neuron process area (dendrite or axon) differ from the spectra of the neuron body: the shape of the 1655 cm^–1^ band indicates that the cell process is dominated by vibrations of double bonds stretching in the fatty acid residues of phospholipids (in terms of position, this band may coincide with the Amide I band associated with vibrations of the peptide bond in proteins, but has a narrower shape), and the 1737 cm^–1^ band shows vibrations of the C=O bonds in the phospholipids. The Raman spectrum band at 1445 cm^–1^ corresponds to vibrations of the methylene groups of lipid molecules with a contribution from vibrations of the same protein groups; the band at 965 cm^–1^ most probably corresponds to vibrations of the phosphate groups in phospholipid molecules and is more pronounced in the process area. It appears that the decrease in the intensity of the Raman spectrum peaks associated with vibrations of the hemoporphyrin bonds of reduced cytochromes is due to a smaller number of mitochondria in the process than in the cell body and, thus, a decrease in the Raman amplitude from the ETC and cytochrome complexes.

The microphotographs of the studied cell culture provide that some cells are localized on the glass surface (see Figure 1 (a)), while other cells have a spherical shape, are not spread out on the glass surface (Figure 1 (b)) and, probably, also differ in their physiological state. Figure 1 (b) shows microphotographs of a spherical neuron and the Raman spectrum recorded in the specified area of the neuron (Figure 1 (c, *3*)). The Raman spectrum maxima at 748, 1125, 1310, 1336, and 1580 cm^–1^ in the cell body area correspond to vibrations of hemes of reduced cytochromes *c* and *b*, the 1448 cm^–1^ band corresponds to vibrations of methylene groups primarily in lipids, whereas 1655 cm^–1^ — to vibrations of Amide I peptide bonds in proteins with alpha structures. It was established that here the Raman spectrum bands corresponding to reduced cytochromes are significantly more intense than in case of a normal fixed cell. This may be due to the filling of the ETC with electrons, which indicates functional disturbances in the cell, apparently in a pre-apoptotic state.

Hence, it can be stated that the presence of characteristic bands corresponding to various types of cytochromes in the Raman spectrum of a neuron allows obtaining information about the state of the mitochondrial respiratory chain, the distribution of mitochondria, proteins, and lipids in the body of neurons and processes. This provides for analysis of the physiological state of the cell and of the development of pathology.

### Study of the conformation of proteins and lipids in the plasma membrane of nerve cells using surface-enhanced Raman spectroscopy with silver nanostructures

**Figure 2.**
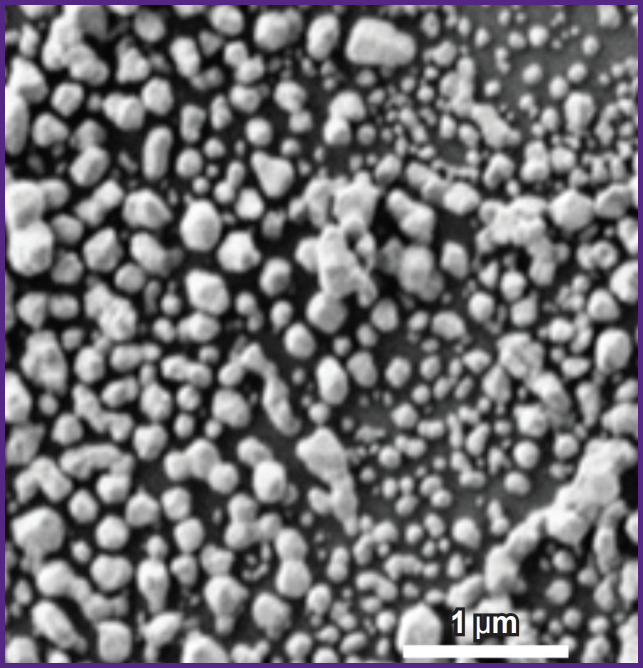
Microphotograph of a silver nanostructured surface taken by using a scanning electron microscope [cited from 4, 15]

**Figure 3.**
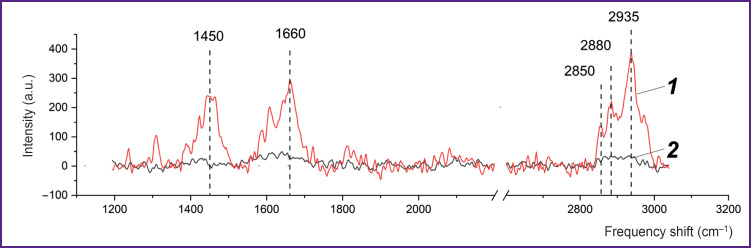
Spectra of a thin neuron process: *1* — SERS spectrum of a thin neuron process placed on a nanostructured surface; *2* — Raman spectrum of the same process on glass without a nanostructured surface. The accumulation time of each spectrum is 120 s, the laser power (532 nm) is up to 1 mW per spot with a diameter of 700 nm

To increase the intensity of the Raman signal recorded from cells and molecules, the researchers used a technical approach based on SERS for highly sensitive and selective study of the conformation of lipids and proteins of the plasma membrane of rat nerve cells, including the plasma membrane of their processes (with diameter of up to 1 μm). Thin processes of nerve cells are transparent and due to the insignificant content of proteins and lipids cannot be studied using traditional RS. The authors demonstrated that under conditions of placing a nerve cell process on a plasmonic silver nanostructured surface (Figure 2) and with laser excitation of 532 nm, the amplification of the Raman amplitude from biomacromolecules located in close proximity to the surface nanostructures being the proteins and lipids of the plasma membrane significantly increases (Figure 3).

Using plasmonic silver nanoparticles, the authors established that the SERS spectrum recorded from the plasma membrane of neurons contains a set of peaks associated with deformation vibrations of CH_2_ methylene groups in lipids and proteins (peak with a maximum position of 1450 cm^–1^), with vibrations of the Amide I band in proteins (1660 cm^–1^), stretching vibrations of C–H bonds of methylene groups in lipids and proteins (peaks 2850 and 2880 cm^–1^), stretching vibrations of C–H bonds of methyl groups in proteins and lipids (2935 cm^–1^) (Figure 3(*1*)). One should note that the Raman spectrum from the membrane molecules of the processes is practically not recorded without silver nanostructures (Figure 3(*2*)).

Thus, the developed approach to the application of SERS for studying neurons can be used to investigate conformational changes in proteins and lipids in the membrane of processes under various impacts (for example, in processes accompanied by the release of a neurotransmitter, a change in the excitability of the process, a change in the content of membrane-bound Ca^2+^ ions, or in pathological changes).

### Study of the distribution and conformation of cytoplasmic proteins and lipids and of the redox state of ETC in mitochondria of astrocytes differentiated from iPSCs of a healthy donor and a patient with Parkinson’s disease

**Figure 4.**
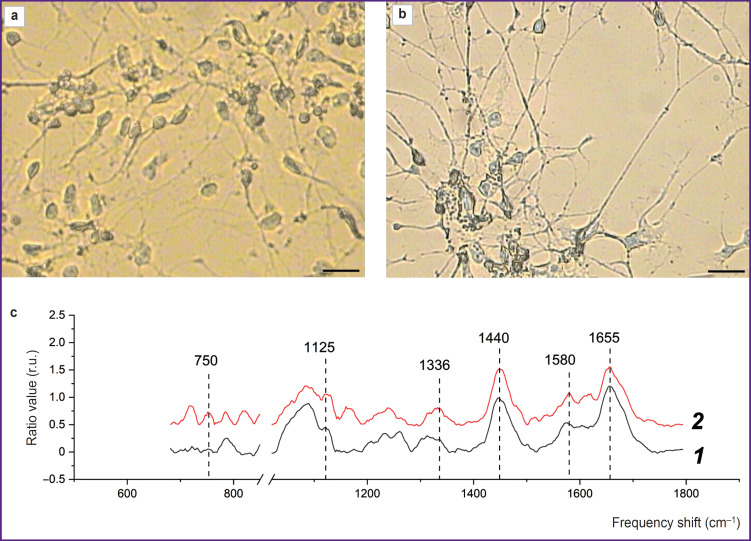
Raman spectra of astrocytes obtained from iPSCs of a healthy donor and a patient with Parkinson’s disease: (a) microphotographs of astrocytes of a healthy donor; (b) microphotographs of astrocytes of a patient with Parkinson’s disease obtained in the video channel of the Raman microspectrometer; bar — 20 μm; (c) Raman spectra of astrocytes obtained from iPSCs of a healthy donor (*1*) and iPSCs of a patient with Parkinson’s disease (*2*). For better clarity, the spectra were normalized to the total intensity of the spectrum for the entire studied spectral range and shifted along the ordinate axis

**Figure 5.**
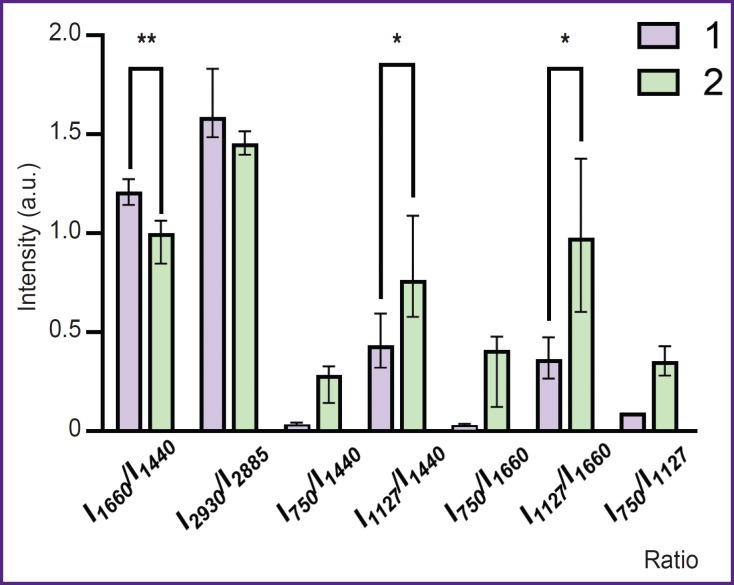
Relative peak intensities in the spectra of astrocytes differentiated from iPSCs of a healthy donor (*1*) and a patient with Parkinson’s disease (*2*) Data are shown as Me [Q1; Q3]; * statistically significant differences, p<0.05; ** p<0.01 according to the Mann–Whitney criterion

The RS technique was used to study the state of the bodies of astrocytes differentiated from iPSCs of a healthy donor and a patient with PD. The maximum Raman signal was detected near the astrocyte nucleus, which may be due to the large number of mitochondria located in this area. The spectra were recorded in the perinuclear area for all studied cells. Figure 4 presents microphotographs of differentiated cells and Raman spectra recorded from astrocytes of a healthy donor and a patient with PD. In both cases, the Raman spectra are characterized by similar peak positions, but the difference is in relative contribution of peaks associated with vibrations of bonds in lipids (peaks with maximum positions of 1440 and 2845 cm^–1^; the high-frequency part of the spectrum is not shown in the Figure 4) and reduced cytochromes *c* and *b* (peaks with maximum positions of 750, 1127, and 1582 cm^–1^). The reduced cytochromes were quantitatively characterized by using the intensity ratios of the bands at 750 and 1440 cm^–1^ (the amount of reduced cytochromes *c* relative to lipids), 1127 and 1440 cm^–1^ (the amount of reduced cytochromes *b* relative to lipids), 750 and 1660 cm^–1^ (the amount of reduced cytochromes *c* relative to protein content), 1127 and 1660 cm^–1^ (the amount of reduced cytochromes *b* relative to protein content) (Figure 5).

It was found that compared to astrocytes of healthy donors in astrocytes of PD patients an increase in the relative intensities of the Raman spectrum peaks associated with vibrations in heme bonds in reduced cytochromes *c* and *b* of mitochondria is seen. This result proves that mitochondria of astrocytes from PD patients contain more cytochromes in the reduced form than mitochondria of astrocytes from healthy donors. This in turn indicates a higher degree of filling the ETC with electrons in the mitochondria of astrocytes in case of pathology. The amount of reduced cytochromes *c* increases compared to the amount of reduced cytochromes *b* (the ratio of the band intensities at 750 and 1127 cm^–1^; see Figure 5). Moreover, the Raman intensity for all peaks associated with vibrations of bonds in lipids — 1440 and 2845 cm^–1^, compared with the peaks associated with vibrations of bonds in proteins 1660 and 2930 cm^–1^, decreases in astrocytes of patients compared to astrocytes of healthy donors. Despite the fact that all of the above mentioned bands are characteristic of both proteins and lipids, they have different intensity in proteins and lipids, and therefore these ratios (ratios of the intensities of the bands at 1660 and 1440 cm^–1^ and 2930 and 2885 cm^–1^) are widely used to assess the relative content of proteins and lipids [29]. Figure 5 demonstrates a decrease in these parameters, which is most probably due to a decrease in the protein content in the cytoplasm and membranes of astrocytes in a patient with PD. These differences may indicate changes in the processes of protein synthesis in the patient’s astrocytes and the accumulation of lipids in the bodies and processes of the patients’ astrocytes.

### Study of the morphology of astrocytes differentiated from iPSCs of a healthy donor and a patient with Parkinson’s disease using scanning ion-conductance microscopy

**Figure 6.**
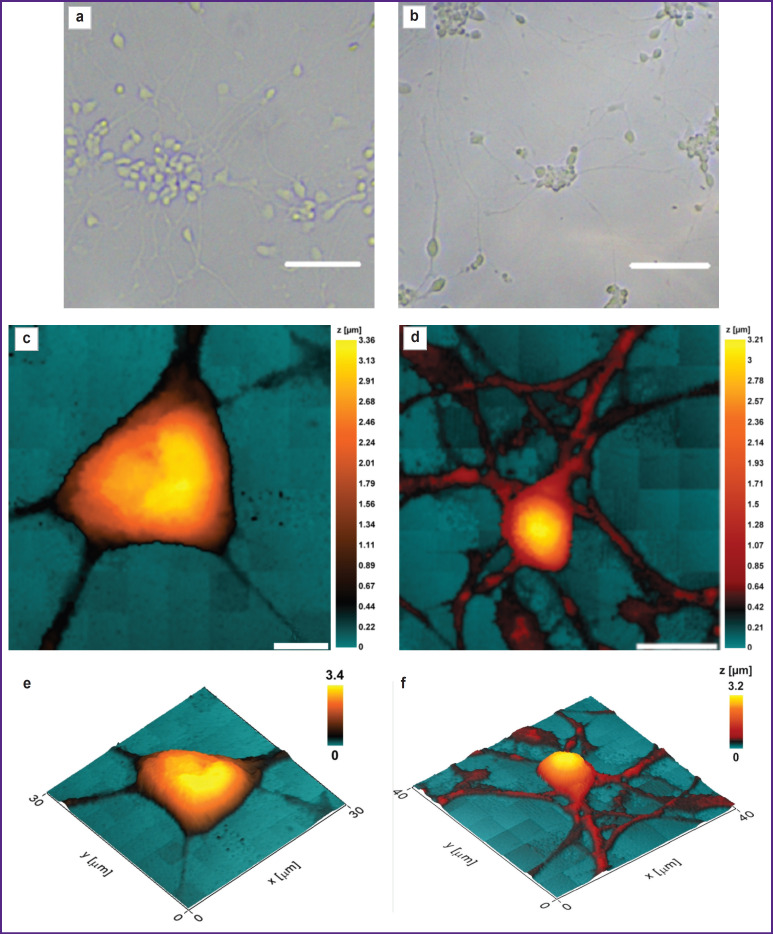
Micrographs and 2D and 3D SICM images of astrocyte bodies and their processes from astrocyte cultures differentiated from iPSCs of a donor and a patient with Parkinson’s disease: (a), (c), (e) astrocytes from iPSCs of a healthy donor; (b), (d), (f) astrocytes from iPSCs of a patient with Parkinson’s disease; (a), (b) micrographs of astrocyte culture (bar — 100 μm). 2D (c), (d) and 3D (e), (f) images of astrocyte bodies were obtained using SICM. The color scale corresponds to the geometric height of the cell

The morphology of cells void of damage or deformation can be studied using the SICM technique. Figure 6 shows microphotographs of astrocyte cultures differentiated using SICM from iPSCs of a healthy donor and a patient with PD.

**Figure 7.**
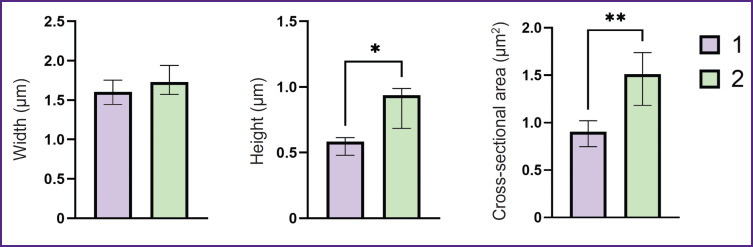
Geometric parameters of astrocyte processes in astrocyte culture differentiated from healthy donor iPSCs (*1*) and from iPSCs of a patient with Parkinson’s disease (*2*) Measurements were made on images obtained using SICM. Data are presented as Me [Q1; Q3]; * p<0.01; ** p<0.001 according to the Mann–Whitney criterion

It was established that the height, as well as the general morphology of the astrocytes bodies obtained from iPSCs of donors and patients with Parkinson’s disease, do not differ significantly, whereas the height of the astrocytes processes obtained from iPSCs of patients with PD is greater than the same value in healthy donors: the height of the processes of astrocytes of healthy donors varies within the range of 0.58 [0.48; 0.61] μm (data are presented as Me [Q1; Q3]), while in astrocytes isolated from cells of a PD patient, the range value was 0.94 [0.68; 0.99] μm. Moreover, the crosssectional area of the processes of astrocytes obtained from iPSCs of PD patients was also significantly higher than the same of astrocytes isolated from the healthy donor culture: the cross-sectional area of the processes of astrocytes isolated from the healthy donor cell culture was 0.90 [0.75; 1.02] μm^2^, while in a PD patient it reached 1.51 [1.18; 1.74] μm^2^ (Figure 7).

The authors assume that such a change in the morphology of the astrocytes processes of PD patients may be associated with changes in protein synthesis and in the protein-lipid composition of the plasma membrane and cytoplasm.

Recently, iPSCs have been used to study the pathogenesis of PD [30, 31], which allows obtaining information on the functioning of human astrocytes without the patients brain biopsy. However, only changes associated with genetic factors are seen in this case, and there are no data on the impact of surrounding neurons and other brain components on astrocytes. There is a known number of astrocyte mutations that are associated with the development of PD and cause a violation of the inflammatory response, the functioning of mitochondria and lysosomes, glutamate uptake, and other functions. It was shown that astrocytes with the LRRK2 G2019S mutation have an impaired ability to form the blood-brain barrier. Such astrocytes also have a number of morphological differences, in particular, the formation of the network of processes is impaired and the number of primary and secondary processes decreases [31]. Mitochondrial functions are also impaired, a decrease in the level of oxygen consumption and ATP production compared to the cells of healthy donors is seen. Apparently, astrocytes switch from oxidative phosphorylation to aerobic glycolysis as the main pathway for energy production. Astrocytes from cells of PD patients contain fewer mitochondria, which are localized primarily in the perinuclear space and are absent in short processes. The level of oxidative damage in astrocytes with PD is higher than in normal cells.

The specified studies are fully consistent with the described picture of impaired morphology and metabolism of astrocytes in PD. According to RS, an increase in the number of reduced cytochromes *c* and *b* is seen in pathology, which indicates a greater ETC loading with electrons. This can lead to increased ROS production. The changes in the morphology of astrocyte processes found by the authors, namely an increase in the height and cross-section of the processes in astrocytes isolated from cell cultures of patients with PD, can be interpreted as a decrease in the relative number of small processes, which is consistent with the available data on the impaired formation of the astrocyte process network in PD [31].

The combination of techniques suggested by the authors allows to obtain more unique and accurate information about the ETC state and the morphology of the astrocyte process network, which provides the improvement of the quality of the PD pathogenesis research at the cellular and molecular levels.

## Conclusion

The study revealed different protein and lipid contents in neuronal bodies and their processes. Wellresolved Raman spectra provide information not only on the composition of different cell areas, but also on conformational changes in their components, which in turn allows to determine the functional state of the cells. The approach using the SERS technique was developed to study thin neuronal processes.

The RS technique allows to detect changes in the relative composition of astrocyte proteins and lipids, as well as the redox state of ETC cytochromes in astrocyte mitochondria induced from pluripotent stem cells of healthy donors and PD patients. It was shown that pathology is accompanied by an increase in the content of reduced cytochromes. This indicates the ETC overreduction, which can lead to excessive production of ROS, causing oxidative stress.

The SICM technique allows obtaining information about the morphology of native cells in culture without their damaging or deformation (which is typical for such techniques as atomic force microscopy).

The combination of RS and SICM first allowed to compare the morphology of astrocyte bodies and processes, as well as the functional state of astrocytes induced from pluripotent stem cells of healthy donors and patients with hereditary Parkinson’s disease.

The results are the basis for creating systems for a comprehensive assessment of the state and functional activity of neurons and astrocytes in various neurodegenerations.
